# Medical Lectures for 1802

**Published:** 1802-09-01

**Authors:** 


### MEDICAL LECTURES for 1802.

The LECTURES given at the Theatres of the contiguous Hospitals of St. Thomas's and Guy's, for 1802, will commence in the following order:

Anatomy and Surgery, by Mr. CLINE, and Mr. ASTLEY COOEPR, on Friday October 1st, at one o'clock.

Practice of Medicine, by Dr. BABINGTON and Dr. CURRY, on Monday, October 4th, at ten in the morning.

Theory and Practice of Chemistry, by Dr. BABINGTON, and Mr. ALLEN, on Tuesday, October 5th, at ten in the morning.

Theory of Medicine and Materia Medica, by Dr. CURRY, on Tuesday October 5, at seven in the evening.

Midwifery, and Diseases of Women and Children, by Dr. HAIGHTON, on Wednesday, October 6th, at eight in the morning.

Physiology, or Laws of the Animal Œconomy, by Dr. HAIGHTON, on Monday, October 11th, at a quarter before seven in the evening.

Principles and Practice of Surgery, by Mr. ASTLEY COOPER, (illustrated by select cases under his care in Guy's Hospital) on Monday October 11th, at eight o'clock in the evening.

☞ The Lectures are so arranged, that no two of them interfere with each other in the hours of attendance; and the whole is calculated to form a complete Course of Medical and Surgical Instruction.

The following Courses of LECTURES will be delivered at the Medical Theatre, St. Bartholomew's Hospital, during the ensuing winter:

On the Theory and Practice of Medicine, by Dr. ROBERTS and Dr. POWELL.

Clinical Lectures on cases occurring in the Hospital will also be given by Dr. ROBERTS.

On Anatomy and Physiology, by Mr. ABERNETHY.

On comparative Anatomy and Physiology, by Mr. MACARTNEY.

On the Theory and Practice of Surgery, by Mr. ABERNETHY.

On Chemstry and the Materia Medica, by Dr. POWELL.

On Midwifery, and the Diseases of Women and Children, by Dr. THYNNE.

The Anatomical Lectures will begin on the 1st of October, at two o'clock, and the other lectures on the succeeding days; which, with other particulars, may be learned by applying to Mr. Nicholson, at the apothecary's shop, St. Bartholomew's Hospital.

THEATRE OF ANATOMY, GREAT WINDMILL STREET.

Mr. WILSON and Mr. THOMAS will begin the Winter Course of their Lectures on Anatomy, Physiology, Pathology, and Surgery, gery, on Friday, the 1st of October, at two o'clock. Practical Anatomy will be continued in the forenoon, as usual. Particulars may be known by applying at the Theatre in Windmill Street.

Mr. Brookes's Autumnal Course of Lectures on Anatomy, Physiology, and Surgery, will commence about the beginning of October, at his Theatre of Anatomy, Blenheim Street.

Mr. Carpue, Surgeon to his Majesty's Forces, will commence his Anatomical Lectures at his Theatre, in Broad Street, Golden Square, on Monday, the 4th of October.

St. Mary-le-bone Infirmary. — Dr. Hooper will give a Course of Lectures on the Theory and Practice of Physic and Morbid Anatomy on Mondays, Wednesdays, and Fridays, at half past six in the evening; and on the Materia Medica, Tuesdays, Thursdays, and Saturdays, at half past seven in the morning.

Dr. Crichton will commence his Autumnal Course of Lectures on the Theory and Practice of Physic, Chemistry, and Materia Medica, on Monday, Oct. 4, 1802.

The Theory and Practice of Physic will be taught every day, except Sunday, at eight o'clock in the morning; the Chemistry every Monday, Wednesday, and Friday, at nine; and the Materia Medica every Tuesday, Thursday, and Saturday, at the same hour.

Dr. Bradley (removed from Delahay Street to Parliament Street) will commence his Autumnal Course of Lectures on the Theory and Practice of Medicine, on Monday, October 4th, at the Lecture-Room, No. 102, Leadenhall Street, at six in the afternoon.

Dr. Wells, one of the Physicians to St. Thomas's Hospital, purposes commencing, in the neighbourhood of that Hospital, on Monday the 4th of October, a Course of Lectures upon the Practice of Physic.

Dr. Rowley intends to give a Course of Lectures in Saville Row, on the Ancient and Modern Medicine, with Aphorisms for Practice.

Dr. Osborn's and Dr. Clarke's Lectures on Midwifery and the Diseases of Women and Children, will in future be read only at Dr. Clarke's House, No. 1, New Burlington Street, Piccadilly.

A Course will begin on Monday, October 4, at a quarter past ten o'clock, and the Lectures will be continued every day at that hour, for the convenience of students attending the Hospitals. Particulars may be known by applying to Dr. Osborn, or to Dr. Clarke, New Burlington Street, Piccadilly.

Dr. Batty's Course of Lectures on the Theory and Practice of Midwifery, will commence on Monday, October 4, 1802, at eleven o'clock in the forenoon, at his house in Great Marlborough Street.

On Friday, October 1, Dr. Dennison and Dr. Squire will begin a Course of Lectures on the Theory and Practice of Midwifery, and the Diseases of Women and Children.

On

On the firft Tuefday evening in October and February, at eight o'clock precifely, Mr. Blair will commence a Courfe of Lectures on Anatomy and Animal Economy. And on the firft Friday evening in October and February, at fix o'clock precifely, he will commence a Courfe of Lectures on the Clinical Practice of Surgery.

On Monday, October 4, at feven o'clock in the evening, the Autumnal Courfe of Lectures on the Principles and Practice of Surgery, will commence in Golden Square, by Mr. John Pearson, Surgeon of the Lock Hofpital, Afylum, &c.

On Monday, October 4, 1802, at feven, P. M. Dr. Pole and Mr. Wagstaff will commence their Autumnal Courfe of Lectures on the Elements and Practice of Midwifery, and Difeafes of Women and Children, at their Lecture Room, No. 102, Leadenhall Street, and the fubfequent Lectures at eight in the evening.

Lectures on Anatomy, Phyfiology, and Surgery, by John Taunton, Member of the Royal College of Surgeons in London, Surgeon to the City Difpenfary, &c.

Thefe Lectures will be given at Mr. Taunton's Lecture Room, No. 35, Hatton Garden; the Winter Courfe to commence on Saturday the 2d day of October, 1802, at eight o'clock in the evening precifely, and be continued every Tuefday, Thurfday, and Saturday at the fame hour. This Courfe will confift of from 30 to 40 Lectures. Tickets two guineas. Further particulars may be known by application to Mr. Taunton, No. 35, Hatton Garden.

Mr. Chevalier, Surgeon to the Weftminfter General Difpenfary, will begin his Autumnal Courfe of Lectures on the Principles and Operations of Surgery, on Monday the 4th of October, at eight o'clock in the evening, at his houfe, in South Audley Street, Grofvenor Square, where printed particulars may be had.

*N. B. If the Notice of any Lectures is omitted, we shall be glad to insert it in our next; and request it may be addressed to the Publisher.*

---

