# Errata

**Published:** 1802-10-01

**Authors:** 


					p. 288, 1. 14, (Dr. Pole's Lecturcs) for Evtiiing, read Morning.

				

